# Genome Sequencing and Organization of Three Geographically Different Isolates of Nucleopolyhedrovirus from the Gypsy Moth Reveal Significant Genomic Differences

**DOI:** 10.2174/0113892029249830231014163829

**Published:** 2023-11-22

**Authors:** Donus Gencer, Cihan Inan, Zeynep Bayramoglu, Remziye Nalcacioglu, Feifei Yin, Zheng Zhu, Jun Wang, Zhihong Hu, Lillian Pavlik, Basil Arif, Zihni Demirbag, Ismail Demir

**Affiliations:** 1 Trabzon University, Salpazarı Vocational School, Department of Property Protection and Security, 61670, Trabzon, Turkey;; 2 Karadeniz Technical University, Faculty of Science, Department of Molecular Biology and Genetics, 61080, Trabzon, Turkey;; 3 Recep Tayyip Erdoğan University, Pazar Vocational School, Department of Plant and Animal Protection, 53330, Rize, Turkey;; 4 Karadeniz Technical University, Faculty of Science, Department of Biology, 61080, Trabzon, Turkey;; 5 Wuhan Institute of Virology, Chinese Academy of Sciences, Wuhan 430071, P.R. China;; 6 Laboratory for Molecular Virology, Great Lakes Forestry Centre, Sault Ste. Marie, Ontario, Canada

**Keywords:** Lymantria dispar multiple nucleopolyhedrovirus, complete genome sequence, geographic isolates, baculovirus, gypsy moth, lymantria dispar

## Abstract

**Background:**

The gypsy moth (*Lymantria dispar* L., Lepidoptera: Erebidae) is a worldwide pest of trees and forests. Lymantria dispar nucleopolyhedrovirus (LdMNPV) belongs to the Baculoviridae family and is an insect virus specific to gypsy moth larvae. In this study, we describe the complete genome sequences of three geographically diverse isolates, H2 (China), J2 (Japan), and T3 (Turkey), of Lymantria dispar multiple nucleopolyhedrovirus (LdMNPV).

**Methods:**

The genomes of isolates H2, J2, and T3 were subjected to shotgun pyrosequencing using Roche 454 FLX and assembled using Roche GS De Novo Assembler. Comparative analysis of all isolates was performed using bioinformatics methods.

**Results:**

The genomes of LdMNPV-H2, J2, and T3 were 164,746, 162,249, and 162,614 bp in size, had GC content of 57.25%, 57.30%, and 57.46%, and contained 162, 165, and 164 putative open reading frames (ORFs ≥ 150 nt), respectively. Comparison between the reference genome LdMNPV-5/6 (AF081810) and the genomes of LdMNPV-H2, J2, and T3 revealed differences in gene content. Compared with LdMNPV-5/6, ORF5, 6, 8, 10, 31, and 67 were absent in LdMNPV-H2, ORF5, 13, and 66 were absent in LdMNPV-J2, and ORF10, 13, 31, and 67 were absent in LdMNPV-T3. In addition, the gene encoding the mucin-like protein (ORF4) was split into two parts in isolates H2 and T3 and designated ORF4a and ORF4b. Phylogenetic analysis grouped isolates H2 and J2 in a different cluster than isolate T3, which is more closely related to the Turkish and Polish isolates. In addition, H2 was found to be closely related to a South Korean LdMNPV isolate.

**Conclusion:**

This study provided a more detailed overview of the relationships between different geographic LdMNPV isolates. The results showed remarkable differences between groups at the genome level.

## 1 INTRODUCTION

Baculoviruses are enveloped particles with double-stranded circular DNA genomes of 80 to 180 kbp. Baculoviruses have been isolated from more than 600 insect species belonging to the orders Lepidoptera, Hymenoptera, and Diptera [1]. They are characterised by being enclosed in a protein structure called an Occlusion Body (OB) at the end of the replication cycle. OBs are usually composed of a highly expressed viral protein (polyhedrin or granulin) that assembles into a protective paracrystalline matrix around the enveloped virions. The family Baculoviridae includes four genera: two of them, *Alphabaculovirus* and *Betabaculovirus*, infect insects of the order Lepidoptera, while the other two, *Gammabaculovirus* and *Deltabaculovirus*, infect insects of the orders Hymenoptera and Diptera, respectively [2]. Alphabaculoviruses are classified into groups I and II based on phylogenetic analysis and the type of fusion glycoprotein in the budded virus envelope. Group I viruses possess GP64, while group II contains F protein in its envelope [3, 4]. To date, 90 baculovirus reference genomes are available in the Genbank database, of which 60 are alphabaculoviruses, 26 are betabaculoviruses, 3 are gammabaculoviruses, and 1 is deltabaculovirus (https://www.ncbi.nlm.nih.gov/genomes/GenomesGroup.cgi?taxid=10442).

The gypsy moth, *Lymantria dispar* (Lepidoptera: Erebidae: Lymantriinae), is a worldwide pest that defoliates forest, fruit, and ornamental trees. The European subspecies, *L. dispar dispar*, is native to Europe, northern Africa, and western Asia and was introduced to North America in the 19^th^ century, where it spread from the northeastern United States to Canada [5]. Over the past 25 years, larvae of the gypsy moth have defoliated an average of 3.0 million forest and shade trees in the northeastern United States, resulting in significant economic losses [6]. *L. dispar* is also a serious pest that occurs in almost all regions of Turkey [7].

Biological control approaches against *L. dispar* consist of mating suppression using gypsy moth pheromones and the application of gypsy moth pathogens [8-11]. The most commonly used pathogens include *Bacillus thuringiensis* subsp. *kurstaki* [12, 13] and the baculovirus *Lymantria dispar multiple nucleopolyhedrovirus* (LdMNPV). Five indigenous isolates of LdMNPV have already been reported from Turkey [14-16]. Many have also been reported in the United States [17, 18], Asia, and Europe [19-25].

Previous studies have evaluated isolates of different geographic origins for their biological activity against the gypsy moth [26, 27]. In terms of virulence, many isolates were less effective than the reference virus (LdMNPV-5/6, a plaque isolate derived from a field isolate from Connecticut, USA, AF081810) [28]. However, two isolates from Japan and China were highly effective against the Asian gypsy moth [29]. LdMNPV has been associated with declines in gypsy moth beetle populations and has been approved as a microbial insecticide in the United States and Canada [28].

The genome of LdMNPV was first sequenced in 1999 and is considered the reference isolate (LdMNPV-5/6) in the NCBI genome database. This isolate contains a 161,046 bp genome with a GC content of 57.5% and is thought to encode 163 proteins [28]. Since only light and electron microscopy data were reported on the first Turkish isolate [14], it is not possible to determine its relationship with the T3 isolate described here.

In the present study, we aim to investigate possible differences between the three local LdMNPV isolates: H2 (China), J2 (Japan), and T3 (Turkey) at the genome level. To uncover the reasons for the possible differences in insecticidal activity, the complete genomes of the isolates were sequenced, annotated, and compared with other baculoviruses.

## 2 MATERIALS AND METHODS

### 2.1 Virus Isolation and Collection

**Fig. (1):**
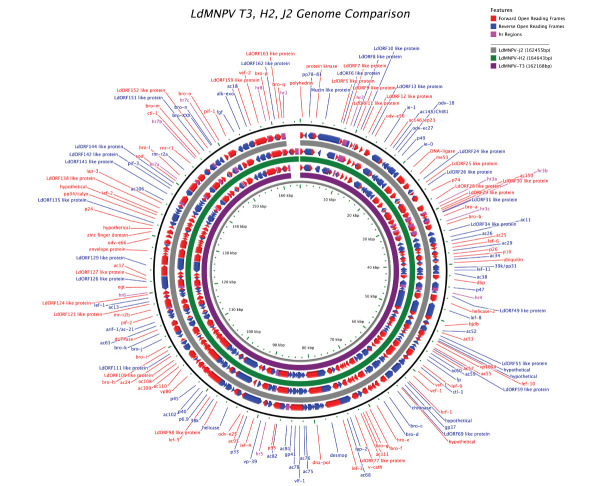
Map showing locations where virus isolates were discovered. The locations of the isolates are marked with red dots.

Genome analysis was performed on three geographic isolates of LdMNPV. Isolates LdMPNV-H2 and -J2 were obtained from the field population of the Asian strain of gypsy moth in Heilongjiang Province of China and Ibaraki Prefecture in Honshu Island of Japan, respectively, while isolate LdMPNV-T3 was isolated from the field population of the European strain of the gypsy moth. LdMNPV-H2, J2, and T3 were collected from Heilongjiang Province of China, Ibaraki Prefecture in Honshu Island of Japan, and Yozgat Province of Turkey, respectively (Fig. **1**).

### 2.2 Virus Purification and DNA Extraction

The three virus isolates were amplified in laboratory-reared larvae of the European strain of the gypsy moth (*Lymantria dispar dispar*). The LdMNPV-H2 and -J2 isolates were prepared as described in Ebling *et al.* [29]. The LdMPV-T3 isolate was also amplified, as described in a previous study [16]. Larvae were homogenized in 0.5% Sodium Dodecyl Sulfate (SDS) in water, and OBs were purified by differential and zonal centrifugation over discontinuous sucrose gradients. OBs were diluted in water, pelleted by centrifugation, and resuspended in water. A solution of 1 M CaCO_3_ and 4 M sodium thioglycolate was added dropwise to the purified OBs, and dissolution was observed by phase-contrast microscopy. The mixture was centrifuged at 5000 rpm for 5 min to remove undissolved OBs, and the supernatant containing virions was loaded onto 10 - 45% (w/ w) cold sucrose gradients prepared in 0.1X TE (1 mM Tris, 0.1 mM EDTA) and centrifuged at 12000 rpm for 30 min in a SW -28 rotor. Sharp virion bands were collected by puncturing the side of the tube, diluted in 0.1X TE, and pelleted at 22000 rpm for 1 hour. The viral pellet was resuspended in TE, and DNA was extracted by standard methods with proteinase K, followed by phenol, phenol/chloroform, and chloroform extractions. The DNA preparations generally had a 260/280 UV absorbance ratio of 1.85.

### 2.3 Sequencing and Bioinformatic Analysis

The genomes of isolates H2, J2, and T3 were subjected to shotgun pyrosequencing with Roche 454 FLX and assembled using Roche GS De Novo Assembler. Sequencing, assembly, gap filling, and identification of repeated sequences were performed as previously described [30]. Hypothetical ORFs and putative ORFs with 50 or more amino acids and minimal overlaps were predicted using Softberry FGENESV0 (http://www.softberry.com/berry.phtml) and NCBI ORF finder (http://www.ncbi.nlm.nih.gov/gorf/gorf.html).

Typical baculovirus (*hr*) homologous regions were identified using the Tandem Repeats Finder (http://tandem.bu.edu/trf/trf.html) [31] and the NCBI BLAST Server (http://blast.ncbi.nlm.nih.gov/Blast.cgi). Predicted ORFs were annotated by homology using NCBI BLAST [32].

Comparative circular genome maps of all isolates were generated using the CGView server (http://wishart.biology.ualberta.ca/cgview/). Secondary structures were predicted using the online server (http://rna.urmc.rochester.edu/RNAstructureWeb/Servers/Predict1/Predict1.html) with default settings for DNA nucleic acid type to draw the structures [33].

### 2.4 Phylogenetic Analysis

A phylogenetic tree was constructed based on the alignments of the nucleotide sequences concatenated using the MEGA10 software with the maximum likelihood method based on the JTT matrix model [34, 35]. LdMNPV-H2, J2, and T3 isolates and seventeen LdMNPV reference isolates were used for phylogenetic analysis. Phylogeny was tested using the bootstrap method with 500 replicates [36].

## 3 RESULTS

### 3.1 Characteristics of the Genomes

Three different geographic isolates of LdMNPV were sequenced and had similar genome size, ORF, and G+C content to the LdMNPV isolates (Table **1**). Whole genome sequence information for all genomes was deposited in GenBank under accession numbers MK264918 (H2), MK089451 (J2), and MF311096 (T3). The total (1.6 Gbp) Q30 (%) base reads of LdNPV-T3, -H2, and -J2 were calculated to be 94.38% (16,.266,888), 91.15% (16,245,722), and 93.42% (16,364,698), respectively.

The sequencing results showed that the H2, J2, and T3 genomes lacked 7, 3, and 5, 133 ORFs, respectively, compared with the LdMNPV-5/6 genome [28] (Table **2**). ORF121 is not present in the genome of T3. In addition, the mucin-like gene (ORF4) was found to be split into two parts in the H2 and T3 genomes, and pp78/81 (ORF2) was found to be divided into two parts in the J2 genome due to point mutations in the genome (Supplementary file **1**).

### 3.2 Phylogenetic Analysis

**Fig. (2):**
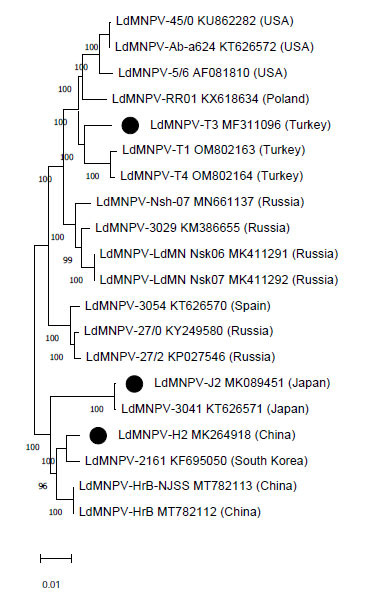
The phylogenetic tree of the newly sequenced isolates of LdMNPV and other LdMNPVs. It was constructed based on the nucleotide sequence alignments of the whole genome using ClusterW and MEGA10. The maximum likelihood phylogram of the isolates is shown with GenBank accession numbers and the origin of the host tested by the bootstrap method with 500 replications. The related alphabaculovirus LyxyNPV-5 from lymantria xylina was included as an outgroup (not shown) [36]. The numbers in the nodes show the percentage bootstrap value. LdMNPV-T3, -H2, and -J2 are marked with a black circle.

The isolate LdMNPV-5/6 was classified into the alphabaculovirus group II, which has greater genomic similarity with LyxyNPV [36]. A maximum likelihood phylogeny based on whole-genome nucleotide sequence alignments is shown in Fig. (**2**) . Mapping of LdMNPV-T3, -H2, and -J2 to the whole genome sequences of the other 17 LdMNPV isolates confirmed a high degree of collinearity between the genomes of these viruses. After phylogeny analysis, isolate LdMNPV-H2 was grouped with isolates LdMNPV-2161 (sequence similarity 98.92%) from South Korea and LdMNPV-HrB-NJSS and -HrB (sequence similarity 98.95% each) from China (Fig. **2**). The isolate LdMNPV-J2 formed a branch with LdMNPv-3041 (sequence similarity 99.85%) from Japan. The isolate LdMNPV-T3 was pooled with isolates from Turkey (LdMNPV-T1/98.48% and -T4/98.77%) and Poland (LdMNPV-RR01/98.51%). Isolates from Russia, Europe and the USA formed a separate group.

### 3.3 Homologous Repeats (*hrs*) and Baculovirus Repeated ORFs (*bros*)

Differences in homologous repeat regions (*hr*) and baculovirus repeated ORFs (*bro* genes) exist between isolates. Alignment of the individual *hr* of the three new isolates with those of LdMNPV-5/6 showed that each *hr* consists of at least one conserved sequence. The data also showed that *hr*8 was absent in H2, and *hr*3c was absent in J2 (Supplementary File **2**). All of these conserved regions contained a core repeat sequence, which has been reported previously [28]. Table **1** presents the genome-wide comparison of our isolates with previously sequenced isolates from different geographic regions by size, ORF, *bro* and *hrs* regions. LdMNPV-H2, -J2 and -T3 isolates have 11, 11 and 13 *hr* regions, respectively.

In this study, *bro* genes were named in alphabetical order and grouped as previously recommended [28] (Table **3**). A notable feature in the genome of LdMNPV is that the *bro* genes and *hr* are linked together to form a larger repeat sequence region (Table **3**). The *bro*-b of the LdMNPV H2 genome is short due to an early termination codon and therefore cannot be classified into defined groups (Table **3**).

## 4 DISCUSSION

This study was performed to determine genome-wide sequence differences between Turkish, Japanese, and Chinese LdMNPV isolates. The genomes of the newly sequenced isolates were annotated and compared with the LdMNPV 5/6 genome [28]. All results are summarized in Tables **1-3** and Supplementary File **1**. Minor genomic alterations in isolates from different parts of the world [21, 29] likely contributed to differential virulence against gypsy moth and/or selective advantage of a particular isolate in different geographic niches.

According to the results, all three isolates contain one copy of the gene encoding FP25K, whereas the LdMNPV-5/6 virus appears to contain FP25K in two parts because the deletion results in a frameshift [28]. This protein plays a role in regulating BV and ODV production, and mutations in the *fp25k* gene appear to be responsible for the few polyhedra (FP) phenomena in AcMNPV [37-39]. The H2 genome carries two genes encoding the viral enhancer factor VEF (VEF-1 and VEF-2), as in the LdMNPV-5/6 genome [28]. The T3 genome has two copies of VEF-1 and one of VEF-2. In comparison, there is one copy of the gene encoding VEF-2 in the J2 genome (Table **2**). Enhancin genes (VEFs) are directly related to NPV virulence. The VEF protein appears to facilitate baculovirus infection by disrupting the peritrophic membrane, allowing virions to access the surface of intestinal cells [40]. Previous studies have shown that deletion of the VEF-1 gene reduces the pathogenicity of this virus twofold [41]. While two of the sequenced genomes have both VEF proteins, the J2 genome lacks *vef*-1, which could affect its ability to infect the host. The *vef*-1 gene is also absent in LdMPNV-3041 (Japan) [22], which is close to our isolate LdMNPV-J2 (Japan) in the phylogenetic analysis of Harrison *et al.* [25]. In addition, the isolates LdMNPV-RR01 (Poland) [42], LdMNPV-Nsk-07 (Russia) (MK411292), and LdMNPV-27/0 (Russia) [43] also lack a *vef*-1 gene. In the NCBI database, the complete genomes of the other isolates, LdMNPV and LdMNPV-J2, were examined, and it was found that the mucin-like protein is one, but the LdMNPV-H2 and LdMNPV-T3 isolates have two of this gene. ORF135 is not present in the genome of LdMNPV-H2 (China), but two other China isolates (LdMNPV-HrB-NJSS and LdMNPV-HrB) contain this gene.

LdORF66, listed as *ctl*-2 [28], encodes a peptide with sequence similarity to snail conotoxins [44]. The genome sequence of J2 (Japan) contains two *ctl* genes, *ctl*-1 (ORF149) and *ctl*-2 (ORF65), similar to the genome of LdMNPV-3041 (Japan) (*ctl*-1, ORF159; *ctl*-2, ORF68). The single *ctl*-1 gene is present in the genomes of isolates LdMNPV-H2 (China, ORF146) and T3 (Turkey, ORF150) and in the genomes of LdMNPV-RR01 (Poland, ORF150), LdMNPV-2161 (South Korea, ORF156), LdMNPV-HrB-NJSS (China, ORF156), and LdMNPV-HrB (China, ORF156).

Homologous regions (*hrs*) were first characterized as enhancer elements and later recognized as origins of DNA replication [45]. Consistent with our observations, it is notable that LdMNPV genomes typically comprise 11 to 13 *hrs* regions, with the noteworthy exception of the Poland isolate, which contains only 6 *hrs* regions. The absence of *hr*7d in our isolates, as well as its absence in isolates RR01 (Poland), 3041 (Japan), and 3029 (Russian) isolates, suggests a possible divergence in the presence of this specific *hr* region among LdMNPV strains. Conversely, the *hr*7d region is consistently present in the genomes of other isolates, including 2161 (South Korea), Ab-624 (Russia), 27/0 (Russia), 6570 (Spain), HrB-NJSS (China), and HrB (China). This variation in the *hr*7d presence underscores the genomic diversity within LdMNPV populations. In addition, our study detected the absence of the *hr*8 region in isolate H2 (China), whereas this region was detected in the genomes of isolates from China (HrB-NJSS and HrB) and South Korea (2161). Although the exact relationship between *hr* counts and infectivity requires further investigation, previous research has demonstrated that the complete removal of *hrs* severely impairs viral DNA replication and progeny production. Conversely, synthetic viruses with *hr*2 or *hr*3 retained the ability to generate subsequent viral generations [46]. Overall, these results highlight the critical role of *hrs* in maintaining viral replication and propagation.

While bioassay studies are required to demonstrate insecticidal activity, the genome structure of an isolate may reveal its potential. Martemyanov *et al.* [41] compared different viral strains against *L. dispar* larvae from different continents. They first used Russian (LdMNPV-27/0) and American (LdMNPV-45/0) strains to evaluate virulence against European and Asian gypsy moth larvae [41]. They concluded that the reason for the differences in virulence was the absence of some *bro* genes and enhancing-encoding genes in the Russian strain. Subsequently, the same group sequenced the genomes of the Russian and American strains and compared their virulence with four other viral strains on European and Asian gypsy moth larvae. The sequencing results showed that the LdMNPV-27/0 strain lacks the enhancing gene *vef-1* and the *bro*-p gene, while the LdMNPV-45/0 lacks the *bro*-e and *bro*-o genes. While the genomes have other sequence differences for some genes, the number of *bro* genes and the missing genes could also have implications for the insecticidal activity of the isolates. Experimental studies have provided insights into the expression and potential functions of bro genes in certain baculoviruses, such as Bombyx mori nucleopolyhedrovirus (BmNPV) and Spodoptera litura multiple nucleopolyhedrovirus [47-50]. However, it is important to note that *bro* genes are absent from the genomes of many other baculoviruses. Some of the *bro* genes in LdMNPV exhibit relatively high conservation, such as the isolate LdMNPV-5/6 ORF72/*bro*-d. However, most bro genes of LdMNPV exhibit limited levels of sequence conservation, suggesting minimal or no functional similarity between the resulting BRO proteins. In this study, we found that the *bro*-b gene in the H2 isolate lacks some residues at the C-terminus. However, two China isolates (HrB-NJSS and HrB) have a *bro*-b gene. In addition, the H2 isolate lacks a *bro* gene compared to the isolates T3 and J2. In previous bioassay studies, the effect of the H2 isolate on Asian and European gypsy moth populations was found to be greater than that of J2 [29]. The absence of the *vef*-1 gene in the genome of J2 may be one of the reasons for its low virulence.

We also found that the newly sequenced LdMNPV isolates from China, Japan, and Turkey were similar to the Korean, Japanese, and Polish isolates, respectively, that had been sequenced previously. Harrison *et al.* [25] performed a phylogenetic analysis of 18 LdMNPV genomes using whole genome nucleotide sequences. In this study, a phylogenetic analysis was performed by adding two LdMNPV isolates (-T1 / OM802163 and -T4 / OM802164) from Turkey and the LdMNPVs used in Harrison *et al.* [25]. The Turkish isolates and the Polish isolates showed a close relationship.

In another study, Harrison *et al.* [22] compared the efficacy of seven geographically isolated LdMNPVs against European and Asian gypsy moth populations and found that the Korean, Russian, and Japanese isolates had stronger insecticidal activity than Gypchek, a commercial product based on an isolate from the USA [22]. The availability of all these isolates makes LdMNPV a good candidate for gypsy moth infestation control.

## CONCLUSION

In conclusion, genome sequencing of baculoviruses is a fundamental tool to increase our knowledge of these viruses and their applications in agriculture, biotechnology, and basic research. It provides valuable insights into their biology, diversity, and potential for practical applications ranging from pest control to protein production. In this study, whole-genome data were obtained from three different geographic isolates and differences were found between them. These differences may also lead to differences in the insecticidal activity of the viruses. In reviewing bioassay studies in the literature, it was found that different geographic isolates may show the same effect, while the same geographic isolates may show different effects. In conclusion, further bioassays with additional isolates and host strains are needed.

## Figures and Tables

**Table 1:** Comparison of genome features of LdMNPVs isolated from different geographical regions.

**Isolate**	**Accession**	**Genome (bp)**	**GC Content**	**ORF Count**	**Hr Count**	**Bro Count**	**References**
LdMNPV-5/6	AF081810	161046	57.5	163	11	16	[28]
LdMNPV-BNP (Poland)	KU377538	157270	50.3	154	6	18	[42]
LdMNPV-3029 (Russia)	KM386655	161712	57.4	168	12	15	[21]
LdMNPV-RR01 (Poland)	KX618634	159729	57.5	166	12	15	[42]
LdMNPV-27/0 (Russia)	KY249580	161727	57.5	160	NA	15	[43]
LdMNPV-45/0 (North America)	KU862282	161880	57.5	157	NA	15	[43]
LdMNPV-27/2 (Russia)	KP027546	164108	57.4	162	NA	18	[51]
LdMNPV-3054 (Spain)	KT626570	164478	57.3	175	13	17	[22]
LdMNPV-3041 (Japan)	KT626571	162658	57.3	178	12	18	[22]
LdMNPV-Ab-a624 (USA)	KT626572	161321	57.5	176	13	15	[22]
LdMNPV 2161 (South Korea)	KF695050	163138	57.3	174	13	20	[20]
HrB-NJSS (China)	MT782113	162282	57.4	174	13	16	[25]
HrB (China)	MT782112	162246	57.4	174	13	16	[25]
H2 (China)	MK264918	164746	57.25	162	11	16	This study
J2 (Japan)	MK089451	162249	57.3	165	11	17	This study
T3 (Turkey)	MF311096	162614	57.46	164	12	17	This study

**Table 2:** Differences between ORFs in LdMNPV genomes related to the reference genome*.

**Reference LdMNPV (AF081810)**	**LdMNPV-H2 (MK264918)**	**LdMNPV-J2 (MK089451)**	**LdMNPV-T3 (MF311096)**
PP78/81 (ORF2)	ORF2	ORF2/ORF3	ORF2
MUCIN-LIKE (ORF4)	ORF4/ORF5	ORF5	ORF4/ORF5
LdORF5	deficiency	deficiency	ORF6
LdORF6	deficiency	ORF6	ORF7
LdORF8	deficiency	ORF8	ORF9
LdORF10	deficiency	ORF10	deficiency
LdORF13	ORF10	deficiency	deficiency
LdORF31	deficiency	ORF30	deficiency
FP25K (ORF63a)	FP25K (ORF60)	FP25K (ORF63)	FP25K (ORF63)
VEF-1 (ORF65)	VEF-1 (ORF62)	deficiency	VEF-1 (ORF65/ORF66)
Ctl-1(ORF66)	deficiency	ORF65	deficiency
LdORF133	deficiency	deficiency	deficiency
ORF121	ORF119	ORF119	deficiency
ORF135	deficiency	ORF133	ORF135

Note:* [28].

**Table 3:** Grouping of bro genes contained in the three LdMNPV isolates and the reference genome.

**-**	**LdMNPV 5/6** **(AF081810)**	**LdMNPV-H2** **(MK264918)**	**LdMNPV-J2** **(MK089451)**	**LdMNPV-T3** **(MF311096)**
**GROUP I**	n	j	b	b
	b	k	i	j
	j	o	j	k
	k	q	l	n
	p	-	m	p
	-	-	n	-
	-	-	o	-
	-	-	q	-
**GROUP II**	a	a	a	a
	l	l	k	l
	m	m	p	m
	o	p	-	o
**GROUP III**	c	c	c	c
	d	d	d	d
	i	e	e	e
	-	f	f	f
	-	-	h	i
**GROUP IV**	e	h	g	g
	f	n	-	h
	g	g	-	-
	h	-	-	-
**Out of Group**	-	b	-	-

## Data Availability

The data that support the findings presented in this study are available within the article and its supplementary information. All sequencing files are available from the NCBI database (https://www.ncbi.nlm.nih.gov/nuccore/MK264918, https://www.ncbi.nlm.nih.gov/nuccore/MK089451, and https://www.ncbi.nlm.nih.gov/nuccore/MF311096).

## LIST OF ABBREVIATIONS

BmNPV: Bombyx mori Nucleopolyhedrovirus
*hrs*: Homologous Regions
LdMNPV: Lymantria dispar Multiple Nucleopolyhedrovirus
OB: Occlusion Body
SDS: Sodium Dodecyl Sulfate
