# Supplementary material for: Genome Sequencing and Organization of Three Geographically Different Isolates of Nucleopolyhedrovirus from the Gypsy Moth Reveal Significant Genomic Differences
Source: Curr Genomics. 2023 Nov 22;24(3):146–54. doi: 10.2174/0113892029249830231014163829 (PMC10761337; doi:10.2174/0113892029249830231014163829)

Current Genomics*, 2023, *Vol. 24, No. 3

Supplementary Material

Genome Sequencing and Organization of Three Geographically Different Isolates of Nucleopolyhedrovirus from the Gypsy Moth Reveal Significant Genomic Differences

Donus Gencer[1], Cihan Inan[2], Zeynep Bayramoglu[3], Remziye Nalcacioglu[4], Feifei Yin[5], Zheng Zhu[5], Jun Wang[5], Zhihong Hu[5], Lillian Pavlik[6], Basil Arif [6], Zihni Demirbag[4] and Ismail Demir[4,*]

[1]Trabzon University, Salpazarı Vocational School, Department of Property Protection and Security, 61670, Trabzon, Turkey; [2]Karadeniz Technical University, Faculty of Science, Department of Molecular Biology and Genetics, 61080, Trabzon, Turkey; [3]Recep Tayyip Erdoğan University, Pazar Vocational School, Department of Plant and Animal Protection, 53330, Rize, Turkey; [4]Karadeniz Technical University, Faculty of Science, Department of Biology, 61080, Trabzon, Turkey; [5]Wuhan Institute of Virology, Chinese Academy of Sciences, Wuhan 430071, P.R. China; [6]Laboratory for Molecular Virology, Great Lakes Forestry Centre, Sault Ste. Marie, Ontario, Canada

Supplementary File 1. ORFs of LdMNPV-H2, -J2 and T3 genomes. ORF lengths are given in nucleotides (nt). ORF amino acid similarities of LdMPNV and LyxyNPV are given for each isolate.

LdMNPV-H2 (MK264918)					LdMNPV-J2 (MK089451)					LdMNPV-T3 (MF311096)				
ORF	Name	Position	No. ORF (& aa identities)		ORF	Name	Position	No. ORF (& aa identities)		ORF	Name	Position	No. ORF (& aa identities)	
			LdMNPV AF081810	LyxyNPV NC_013953				LdMNPV AF081810	LyxyNPV NC_013953				LdMNPV AF081810	LyxyNPV NC_013953
1	polyhedrin	1>738	1 (100)	1 (100)	1	polyhedrin	1>738	1 (100)	1 (100)	1	polyhedrin	1>738	1 (100)	1 (100)
2	pp78/81	746<2389	2 (93.4)	2 (72.5)	2	pp78/81	746<1240	2 (99.3)	2 (87.2)	2	pp78-81	746<2311	2 (92.4)	2 (70.5)
					3	pp78/81	1465<2400	2 (93.4)	2 (68.9)					
3	pk-1	2391>3215	3 (98.9)	3 (87.9)	4	pk-1	2402> 3226	3 (98.9)	3 (87.9)	3	Protein kinase	2313>3137	3 (99.6)	3 (87.9)
4	mucin like	3838<4446	4 (94.5)	4 (57)	5	mucin like	3829< 7494	4 (84.4)	4 (69.3)	4	Mucin like protein	3730<4941	4 (89)	4 (41)
5	mucin like	5808<7631	4 (88.3)	4 (42)						5	Mucin like protein	5478<7346	4 (91)	4 (69)
										6	LdORF5 like protein	6774>7343	5 (98.4)	5 (62)

LdMNPV-H2 (MK264918)					LdMNPV-J2 (MK089451)					LdMNPV-T3 (MF311096)				
ORF	Name	Position	No. ORF (& aa identities)		ORF	Name	Position	No. ORF (& aa identities)		ORF	Name	Position	No. ORF (& aa identities)	
			LdMNPV AF081810	LyxyNPV NC_013953				LdMNPV AF081810	LyxyNPV NC_013953				LdMNPV AF081810	LyxyNPV NC_013953
					6	LdORF6 like protein	7482< 7673	6 (100)		7	LdORF6 like protein	7334<7585	6 (72.8)	
6	LdORF7 like protein	7925>8467	7 (98.8)	6 (86.6)	7	LdORF7 like protein	7768> 8316	7 (98.3)	6 (86.5)	8	LdORF7 like protein	7680>8222	7 (99.4)	7 (42.8)
	8471-9715					8318-9191					hr2	8560-8946		
					8	LdORF8 like protein	9193< 9606	8 (90.6)		9	LdORF8 like protein	9008<9589	8 (68.6)	
7	LdORF9 like protein	9719>10282	9 (93)		9	LdORF9 like protein	9920> 10423	9 (87.7)		10	LdORF9 like protein	9682>10200	9 (98.2)	
					10	LdORF10 like protein	10560< 10670	10 (83.3)						
8	LdORF11 like protein	10725>11489	11 (85.5)		11	LdORF11 like protein	10832> 11596	11 (84.7)		11	LdORF11 like protein	10643>11407	11 (87.5)	
9	LdORF12 like protein	11882>12412	12 (91)		12	LdORF12 like protein	11994> 12530	12 (86.5)		12	LdORF12 like protein	11787>12338	12 (90.2)	
10	LdORF13 like protein	12612<12749	13 (94.7)											
11	odv-e56	12968>14038	14 (98.8)	10 (98)	13	odv-e56	13053> 14123	14 (98.8)	10 (98)	13	odv-e56	12763>13833	14 (99.1)	10 (98.3)
12	ie1	14510<16201	15 (97.1)	12 (81.6)	14	ie1	14602< 16293	15 (97)	12 (81.4)	14	ie-1	14325<16016	15 (97.5)	12 (81.6)
13	ac146	16258>16881	16 (95.6)	13 (83.3)	15	ac146	16350> 16991	16 (93.6)	13 (81.8)	15	ac146/ep23	16073>16699	16 (99.5)	13 (81.4)
14	ac145	16969<17247	17 (100)	14 (85.8)	16	ac145	17137< 17415	17 (98.9)	14 (85.8)	16	ac145/ChtB1	16804<17082	17 (100)	14 (85.8)
15	odv-e27	17260<18111	18 (99.2)	15 (84.6)	17	odv-e27	17428< 18279	18 (99.2)	15 (84.6)	17	odv-ec27	17095<17946	18 (99.6)	15 (84.6)
16	odv-e18	18129<18395	19 (100)	16 (98.8)	18	odv-e18	18297< 18563	19 (100)	16 (98.8)	18	odv-18	17964<18230	19 (100)	16 (98.8)
17	ac142/p49	18367<19818	20 (99.7)	17 (94.2)	19	ac142/p49	18535< 19986	20 (99.7)	17 (94.2)	19	p49	18202<19653	20 (99.5)	17 (94)
18	ie-0	19815<20591	21 (98.8)	18 (90.3)	20	ie-0	19983< 20792	21 (98.4)	18 (90.3)	20	ie-0	19650<20426	21 (98.8)	18 (89.9)
19	DNA-ligase	21040>22689	22 (97.2)	20 (80.5)	21	DNA-ligase	21208> 22857	22 (97)	20 (80.5)	21	DNA-ligase	20855>22501	22 (100)	20 (80.6)
20	Me53	22970>23995	23 (99.4)	21 (88.8)	22	me53	23141> 24166	23 (99.1)	21 (88.5)	22	me53	22781>23809	23 (100)	21 (88.2)
21	LdORF24	24232<24858	24 (97.6)		23	LdORF24	24380< 25006	24 (97.1)		23	LdORF24	24031<24657	24 (99.5)	

LdMNPV-H2 (MK264918)					LdMNPV-J2 (MK089451)					LdMNPV-T3 (MF311096)				
**ORF**	**Name**	**Position**	**No. ORF (& aa identities)**		**ORF**	**Name**	**Position**	**No. ORF (& aa identities)**		**ORF**	**Name**	**Position**	**No. ORF (& aa identities)**	
			LdMNPV AF081810	LyxyNPV NC_013953				LdMNPV AF081810	LyxyNPV NC_013953				LdMNPV AF081810	LyxyNPV NC_013953
	like protein					like protein					like protein			
22	LdORF25 like protein	25031>25495	25 (96.7)	22 (73.5)	24	LdORF25 like protein	25179> 25643	25 (96.7)	22 (73.5)	24	LdORF25 like protein	24830>25294	25 (99.3)	22 (74.1)
23	LdORF26 like protein	25724<25942	26 (98.6)	23 (81.9)	25	LdORF26 like protein	25877< 26095	26 (98.6)	23 (81.9)	25	LdORF26 like protein	25526<25744	26 (100)	23 (80.5)
24	p74	25954>27972	27 (98.9)	24 (95.5)	26	p74	26107> 28125	27 (98.8)	24 (95.9)	26	p74	25756>27774	27 (99.2)	24 (95.8)
25	LdORF28 like protein	28020>29099	28 (77.7)	25 (77.4)	27	LdORF28 like protein	28173> 29297	28 (94.6)	25 (89.3)	27	LdORF28 like protein	27822>28961	28 (98.6)	25 (90.1)
	hr3a	29103-29846				hr3a	29551-29962				hr3a	29015-29524		
26	LdORF29 like protein	29851>30297	29 (87.8)	26 (91.4)	28	LdORF29 like protein	29964> 30410	29 (85.8)	26 (89.3)	28	LdORF29 like protein	29569>30015	29 (87.8)	26 (89.8)
27	ac150	30392>30676	30 (91.4)	27 (94.6)	29	ac150	30504> 30788	30 (90.4)	27 (93.6)	29	LdORF30 like protein	30109>30393	30 (98.9)	27 (91.4)
	hr3b	30679-31309				hr3b	30977-31698				hr3b	30642-30938		
					30	LdORF31 like protein	30835<30975	31 (68.7)						
28	bro-a	31314>32366	32 (91.7)	28 (93.4)	31	bro-a	31700>31978	32 (86.8)	28 (74.7)	30	bro-a	31024>32076	32 (92.8)	28 (89.1)
	hr3c	32371-33148									hr3c	32134-32985		
29	bro-b	33154>33600	33 (94.1)	29 (76.1)	32	bro-b	32163>33209	33 (91)	29 (76.1)	31	bro-b	32594>33565	33 (98.1)	29 (80.8)
30	LdORF34 like protein	33876<34676	34 (96.3)	30 (92.3)	33	LdORF34 like protein	33504<34265	34 (96.8)	30 (91.3)	32	LdORF34 like protein	33837<34598	34 (100)	30 (90.9)
31	ac11	34822<35907	35 (95.5)	31 (89.9)	34	ac11	34472<35557	35 (95.5)	31 (89.9)	33	ac11	34757<35836	35 (99.1)	31 (89.3)
32	ac26	35841<36212	36 (99.1)	32 (86.9)	35	ac26	35491<35862	36 (97.5)	32 (86.9)	34	ac26	35770<36141	36 (100)	32 (87.8)
33	ac25	36257>36976	37 (99.1)	33 (84.5)	36	ac25	35907>36626	37 (98.7)	33 (84.1)	35	ac25	36186>36902	37 (98.7)	33 (84.8)
34	lef-6	36983>37459	38 (98.1)	34 (63)	37	lef-6	36633>37115	38 (98.7)	34 (64.2)	36	lef-6	36909>37382	38 (98.7)	34 (64.2)
35	ac29	37647<37853	39 (98.5)	35 (95.5)	38	ac29	37315<37521	39 (98.5)	35 (95.5)	37	ac29	37578<37784	39 (100)	35 (94.1)
36	p26	37933>38691	40 (98.4)	36 (76.9)	39	p26	37601>38359	40 (98)	36 (76.5)	38	p26	37864>38622	40 (99.6)	36 (76.1)

**LdMNPV-H2 (MK264918)**					**LdMNPV-J2 (MK089451)**					**LdMNPV-T3 (MF311096)**				
**ORF**	**Name**	**Position**	**No. ORF (& aa identities)**		**ORF**	**Name**	**Position**	**No. ORF (& aa identities)**		**ORF**	**Name**	**Position**	**No. ORF (& aa identities)**	
			LdMNPV AF081810	LyxyNPV NC_013953				LdMNPV AF081810	LyxyNPV NC_013953				LdMNPV AF081810	LyxyNPV NC_013953
37	p10	38726>38959	41 (100)		40	p10	38394>38627	41 (100)		39	p10	38657>38890	41 (100)	
38	ac34	38963<39529	42 (99.4)	38 (86.7)	41	ac34	38631<39197	42 (98.9)	38 (86.7)	40	ac34	38894<39460	42 (98.9)	38 (86.2)
39	ubiquitin	39607>40059	43 (96.6)	39 (82.1)	42	ubiquitin	39275>39727	43 (96)	39 (81.4)	41	ubiquitin	39538>39990	43 (98.6)	39 (82.7)
40	39k/pp31	40231<41025	44 (100)	40 (84.8)	43	39k/pp31	39899<40690	44 (99.2)	40 (84.7)	42	39k/pp31	40171<40965	44 (100)	40 (84.8)
41	lef-11	40814<41377	45 (98.9)	41 (81.7)	44	lef-11	40479<41042	45 (98.4)	41 (81.1)	43	lef-11	40754<41317	45 (99.4)	41 (81.7)
42	ac38	41296<42048	46 (98.4)	42 (93.6)	45	ac38	40961<41713	46 (98.4)	42 (93.6)	44	ac38	41236<41979	46 (100)	42 (92)
43	dbp	42259>43185	47 (96.8)	43 (95.7)	46	dbp	41924>42850	47 (97.2)	43 (95.4)	45	dbp	42189>43115	47 (99.6)	43 (95.7)
44	p47	43543<44715	48 (99.4)	44 (96.9)	47	p47	43208<44380	48 (99.4)	44 (96.9)	46	p47	43438<44610	48 (99.7)	44 (96.6)
	hr4	44719-45290				hr4	44382-45004				hr4	44681-45262		
45	LdORF49 like protein	45294<45689	49 (88.3)		48	LdORF49 like protein	45005<45325	49 (92.4)		47	LdORF49 like protein	45325<45687	49 (88.2)	
46	helicase-2	45967>47343	50 (97.8)	47 (98)	49	helicase-2	45758>47134	50 (97.8)	47 (98)	48	helicase-2	46052>47434	50 (99.7)	47 (97.8)
47	lef-8	47543<50158	51 (98.8)	48 (94.5)	50	lef-8	47333<49951	51 (98.7)	48 (94.2)	49	lef-8	47599<50226	51 (99.2)	48 (94)
48	bjdb	50157>51032		49 (73.6)	51	bjdb	49950>50816		49 (72.3)	50	bjdb	50225>51127		49 (74.1)
49	ac52	51066<51755	53 (97.8)	50 (74.5)	52	ac52	50852<51541	53 (98.2)	50 (75.1)	51	ac52	51162<52064	53 (100)	50 (75.1)
50	ac53	51646>52074	54 (97.8)	52 (91.5)	53	ac53	51432>51860	54 (97.1)	52 (90.8)	52	ac53	51742>52170	54 (99.3)	52 (91.5)
51	LdORF55 like protein	52082<53164	55 (82.5)	53 (61.2)	54	LdORF55 like protein	51867<52946	55 (81.7)	53 (60.7)	53	LdORF55 like protein	52220<52855	55 (96.6)	53 (67.7)
52	hypothetical	53173<53409			55	hypothetical	52955< 53191			54	hypothetical	52903<53316		
										55	hypothetical	53325<53561		
53	lef-10	53342>53596	56 (97.3)	54 (91.6)	56	lef-10	53124>53378	56 (97.3)	54 (91.6)	56	lef-10	53518>53748	56 (100)	54 (93.4)
54	vp1054	53457>54455	57 (98.1)	55 (91.8)	57	vp1054	53239>54237	57 (98.1)	55 (91.8)	57	vp1054	53609>54607	57 (99.7)	55 (91.5)
55	ac55	54500>54769	58 (98.4)	56 (84.4)	58	ac55	54282>54551	58 (98.4)	56 (84.4)	58	ac55	54731>54925	58 (100)	56 (90.6)
56	LdORF59 like protein	54860<55021	59 (98.1)		59	LdORF59 like protein	54642<54803	59 (98.1)		59	LdORF59 like protein	55019<55180	59 (100)	

LdMNPV-H2 (MK264918)					LdMNPV-J2 (MK089451)					LdMNPV-T3 (MF311096)				
**ORF**	**Name**	**Position**	**No. ORF (& aa identities)**		**ORF**	**Name**	**Position**	**No. ORF (& aa identities)**		**ORF**	**Name**	**Position**	**No. ORF (& aa identities)**	
			LdMNPV AF081810	LyxyNPV NC_013953				LdMNPV AF081810	LyxyNPV NC_013953				LdMNPV AF081810	LyxyNPV NC_013953
57	ac57	55023>55505	60 (93.2)	57 (87.5)	60	ac57	54805>55287	60 (93.2)	57 (88.1)	60	ac57	55182>55676	60 (99.3)	57 (87.8)
58	ChaB-like peptide ac58/ac59	55488<56030	61 (65.4)	58 (55.3)	61	ChaB-like peptide ac58/ac59	55270<55821	61 (70.6)	58 (55)	61	ac59	55659<56231	61 (98.9)	58 (55.8)
59	ac60	56283<56588	62 (94.2)	59 (75)	62	ac60	56073<56372	62 (94.2)	59 (76.5)	62	ac60	56436<56726	62 (95.8)	59 (83.1)
60	fp	56704<57366	63a (100)	60 (84.5)	63	fp	56493<57155	63a (100)	60 (84.5)	63	fp	56854<57507	63a (95.7)	60 (84.7)
61	lef-9	57475>58965	64 (98.7)	61 (95.1)	64	lef-9	57264>58751	64 (98.7)	61 (94.5)	64	lef-9	57626>59116	64 (99.4)	61 (94.9)
62	vef-1	59017>61368	65 (96.8)	62 (90.3)						65	vef-1	59168>60202	65 (98.2)	62 (84.3)
										66	vef-1	60120>61517	65 (97.6)	62 (89.3)
					65	ctl-2	58792<58953	66 (86.6)	63 (94.3)					
63	hrf-1	61489>62145	67 (94.5)		66	hrf-1	58989>59645	67 (95.8)		67	hrf-1	61640>62296	67 (99)	
64	hypothetical	62183<62362			67	hypothetical	59687<59866							
65	gp37	62352<63161	68 (98.1)	64 (90.7)	68	gp37	59856<60665	68 (97.4)	64 (90.4)	68	gp37	62485<63294	68 (99.6)	64 (91.5)
66	LdORF69 like protein	63417<63569	69 (96)		69	hypothetical	60707>61393		65 (87.5)	69	hypothetical	63336>64022		65 (85)
67	chitinase	63570<65246	70 (99.6)	66 (97.8)	70	chitinase	61074<62750	70 (99.4)	66 (97.6)	70	chitinase	63703<65379	70 (99.8)	66 (97.6)
68	bro-c	65381<66877	71 (73.2)		71	bro-c	62885< 64432	71 (69.2)		71	bro-c	65514<67046	71 (90.7)	
69	bro-d	66894<67286	71 (84.7)		72	bro-d	64734>65915	72 (91.4)		72	bro-d	67049<67846	71 (40.4)	
70	bro-e	67792>69339	72 (94.1)		73	bro-e	66429>67190	74 (96.9)		73	bro-e	68087>69619	72 (95.4)	
71	bro-f	69494>70255	73/74 (91.3)		74	bro-f	67263>67931	75 (92.7)		74	bro-f	69779>70561	74 (96.9)	
	bro-g	70328>70996	75 (96.4)							75	bro-g	70634>71302	75 (99.1)	
72	ac111	71028>71297	76 (95.5)	72 (78.6)	75	ac111	67963>68229	76 (96.5)	72 (80.6)	76	ac111	71334>71603	76 (96.6)	72 (76.4)
73	LdORF77 like protein	71248>71871	77 (92.7)	73 (90.8)	76	LdORF77 like protein	68180>68803	77 (92.7)	73 (90.8)	77	LdORF77 like protein	71554>72198	77 (98.6)	73 (88.9)
74	v-cath	72068>73150	78 (98)	74 (93.4)	77	v-cath	69000>70082	78 (98)	74 (93.4)	78	v-cath	72374>73456	78 (98.3)	74 (93.7)

LdMNPV-H2 (MK264918)					LdMNPV-J2 (MK089451)					LdMNPV-T3 (MF311096)				
ORF	Name	Position	No. ORF (& aa identities)		ORF	Name	Position	No. ORF (& aa identities)		ORF	Name	Position	No. ORF (& aa identities)	
			LdMNPV AF081810	LyxyNPV NC_013953				LdMNPV AF081810	LyxyNPV NC_013953				LdMNPV AF081810	LyxyNPV NC_013953
75	iap-2	73153<73860	79 (98.3)	75 (77)	78	iap-2	70085<70786	79 (97)	75 (78.5)	79	iap-2	73459<74163	79 (99.1)	75 (76.5)
76	ac68	73857<74246	80 (96.6)	76 (89.8)	79	ac68	70783<71172	80 (95.2)	76 (90.6)	80	ac68	74160<74567	80 (97.6)	76 (87.3)
77	lef-3	74245>75366	81 (97)	77 (83.1)	80	lef-3	71171>72292	81 (97.3)	77 (83.3)	81	lef-3	74566>75687	81 (98.6)	77 (84.4)
78	desmop	75558<77879	82 (95.1)	78 (68.4)	81	desmop	72482<74794	82 (95.1)	78 (68.4)	82	desmop	75941<78244	82 (98.9)	78 (69.4)
79	dna-pol	77761>80796	83 (98.3)	79 (88.1)	82	dna-pol	74676>77711	83 (98.3)	79 (88.1)	83	dna-pol	78126>81173	83 (98.9)	79 (88.6)
80	ac75	80945<81331	84 (100)	80 (88.2)	83	ac75	77863<78249	84 (100)	80 (88.2)	84	ac75	81327<81713	84 (100)	80 (88.2)
81	ac76	81333<81593	85 (100)	81 (97.6)	84	ac76	78251<78511	85 (100)	81 (97.6)	85	ac76	81715<81975	85 (100)	81 (97.6)
82	vlf-1	81643<82779	86 (99.2)	82 (91.7)	85	vlf-1	78561<79697	86 (99.2)	82 (91.7)	86	vlf-1	82025<83161	86 (99.4)	82 (91.7)
83	ac78	82793<83128	87 (94.6)	83 (65.7)	86	ac78	79711<80046	87 (94.6)	83 (65.7)	87	ac78	83175<83507	87 (95.5)	83 (66)
84	gp41	83125<84096	88 (100)	84 (96.8)	87	gp41	80043<81014	88 (100)	84 (96.8)	88	gp41	83504<84475	88 (100)	84 (96.8)
85	ac81	84077<84736	89 (98.1)	85 (88.8)	88	ac81	80995<81663	89 (98.1)	85 (88.8)	89	ac81	84456<85124	89 (98.1)	85 (88.9)
86	ac82	84621<85292	90 (97.3)	86 (77.9)	89	ac82	81539<82219	90 (97.3)	86 (77.9)	90	ac82	85000<85680	90 (97.7)	86 (77.9)
87	p95	85162>87732	91 (89.2)	87 (79.6)	90	p95	82089>84608	91 (89.2)	87 (79.6)	91	p95	85550>88057	91 (95.1)	87 (81.2)
	hr5	87737-88561				hr5	84610-85263				hr5	88099-88678		
88	vp39	88568<89561	92 (99.3)	88 (92.7)	91	vp39	85265<86341	92 (99.3)	88 (92.7)	92	vp39	88782<89837	92 (99.1)	88 (91.6)
89	lef-4	89631>91088	93 (98.1)	89 (88)	92	lef-4	86340>87797	93 (98.1)	89 (88)	93	lef-4	89836>91293	93 (99.7)	89 (87.4)
90	ac92/p33	87915<88670	94 (100)	90 (88.4)	93	ac92/p33	87915<88670	94 (100)	90 (88.4)	94	p33	91411<92166	94 (98.8)	90 (89.6)
91	ac93	91206<91961	95 (100)	91 (96.2)	94	ac93	88669>89148	95 (100)	91 (96.2)	95	ac93	92165>92644	95 (98.1)	91 (98.1)
92	odv-e25	92441>93094	96 (98.6)	92 (95.3)	95	odv-e25	89150>89803	96 (98.6)	92 (95.3)	96	odv-e25	92646>93299	96 (99)	92 (94.9)
93	helicase	93317<96988	97 (99.1)	93 (91.1)	96	helicase	90026<93697	97 (99.1)	93 (91.1)	97	helicase	93539<97246	97 (98)	93 (90.7)
94	LdORF98 like protein	96945>97466	98 (100)	94 (91.9)	97	LdORF98 like protein	93654>94175	98 (100)	94 (91.9)	98	LdORF98 like protein	97203>97724	98 (99.4)	94 (91.9)
95	38k	97460<98428	99 (97.8)	95 (89.7)	98	38k	94169<95137	99 (97.8)	95 (89.7)	99	38k	97718<98686	99 (98.7)	95 (90.3)
96	lef-5	98321>99157	100 (98.2)	96 (90.6)	99	lef-5	95030>95866	100 (98.2)	96 (90.6)	100	lef-5	98579>99205	100 (98)	96 (90.5)
97	p6.9	99151<99462	101 (95.1)	97 (88.8)	100	p6.9	95860<96174	101 (95.1)	97 (88.8)	101	p6.9	99408<99716	101 (97)	97 (88.8)

LdMNPV-H2 (MK264918)					LdMNPV-J2 (MK089451)					LdMNPV-T3 (MF311096)				
**ORF**	**Name**	**Position**	**No. ORF (& aa identities)**		**ORF**	**Name**	**Position**	**No. ORF (& aa identities)**		**ORF**	**Name**	**Position**	**No. ORF (& aa identities)**	
			LdMNPV AF081810	LyxyNPV NC_013953				LdMNPV AF081810	LyxyNPV NC_013953				LdMNPV AF081810	LyxyNPV NC_013953
98	p40	99660<100805	102 (100)	98 (91.6)	101	p40	96372<97517	102 (100)	98 (91.6)	102	p40	99902<101047	102 (100)	98 (91.6)
99	ac102	100825<101190	103 (99.1)	99 (94.2)	102	ac102	97530<97895	103 (99.1)	99 (94.2)	103	ac102	101067<101432	103 (100)	99 (95)
100	p45	101183<102352	104 (98.7)	100 (90.7)	103	p45	97888<99057	104 (98.7)	100 (90.7)	104	p45	101425<102594	104 (99.4)	100 (91)
101	vp80	102376>105261	105 (96.4)	101 (71.8)	104	vp80	99081>101969	105 (96.4)	101 (71.8)	105	vp80	102618>105557	105 (97.5)	101 (69.3)
102	ac110	105263>105433	106 (98.2)	102 (92.7)	105	ac110	101971>102141	106 (98.2)	102 (92.7)	106	ac110	105559>105729	106 (100)	102 (90.9)
103	ac109	105439>106539	107 (99.7)	103 (91.2)	106	ac109	102147>103247	107 (99.7)	103 (91.2)	107	ac109	105735>106835	107 (100)	103 (91)
104	ac108	106542>106832	108 (92.7)	104 (80.2)	107	ac108	103250>103540	108 (92.7)	104 (80.2)	108	ac108	106838>107131	108 (98.9)	104 (82.4)
105	LdORF109 like protein	106866>107867	109 (97.2)	105 (87.6)	108	LdORF109 like protein	103574>104575	109 (97.8)	105 (87.6)	109	LdORF109 like protein	107165>108169	109 (99)	105 (87.3)
106	ac24	107953>108492	110 (99.4)	106 (94.9)	109	ac24	104659>105198	110 (99.4)	106 (94.9)	110	ac24	108241>108780	110 (99.4)	106 (94.9)
107	LdORF111 like protein	108517<108819	111 (98)	107 (84)	110	LdORF111 like protein	105223<105525	111 (98)	107 (84)	111	LdORF111 like protein	108805<109107	111 (100)	107 (84)
108	bro-h	109297>109590	113 (56.9)		111	bro-g	105785>106078	112 (86)		112	bro-h	109474>109743	112 (92.8)	108 (31.8)
109	bro-i	109621>111198	113 (64.3)		112	bro-h	106011>107435	113 (84.8)		113	bro-i	109638>111128	113 (90.9)	108 (34.6)
110	bro-j	111232<112482	114 (90.1)		113	bro-i	107579<108826	114 (87.9)		114	bro-j	111162<112406	114 (88.8)	142 (53.6)
111	bro-k	112538<113260	115 (88.8)		114	bro-j	108883<109605	115 (87.9)		115	bro-k	112461<113177	115 (96.2)	145 (46.5)
112	dUTPase	113419>113868	116 (99.3)	141 (96.6)	115	dUTPase	109764>110213	116 (99.3)	141 (96.6)	116	dUTPase	113344>113793	116 (99.3)	141 (96.6)
113	ac63	114117<114581	117 (95.4)	140 (64.5)	116	ac63	110482<110946	117 (95.4)	140 (64.5)	117	ac63	114010<114474	117 (99.3)	140 (64.5)
114	arif-1/ac21	114633<115433	118 (97)	139 (85.9)	117	arif-1/ac21	110999<111799	118 (97)	139 (85.9)	118	arif-1/ac21	114527<115336	118 (100)	139 (84.5)
115	pif-2	115473>116696	119 (99.5)	138 (94.3)	118	pif-2	111839>113062	119 (99.2)	138 (94.1)	119	pif-2	115376>116599	119 (99.5)	138 (94.3)
116	rnr-r2b	116718>117797	120 (96.3)	137 (93.9)	119	rnr-r2b	113084>114163	120 (96.1)	137 (93.6)	120	rnr-r2b	116621>117640	120 (97.1)	137 (93.4)
117	Lyxy136 like protein	118196<118402		136 (66.6)	120	Lyxy136 like protein	114562< 114768		136 (66.7)					
										121	LdORF121 like protein	118020>118256	121 (98.7)	
118	ac13	118404<119012	122 (97.5)	135 (68.9)	121	ac13	114770<115381	122 (98)	135 (69.4)	122	ac13	118253<118864	122 (98)	135 (68.9)

LdMNPV-H2 (MK264918)					LdMNPV-J2 (MK089451)					LdMNPV-T3 (MF311096)				
ORF	Name	Position	No. ORF (& aa identities)		ORF	Name	Position	No. ORF (& aa identities)		ORF	Name	Position	No. ORF (& aa identities)	
			LdMNPV AF081810	LyxyNPV NC_013953				LdMNPV AF081810	LyxyNPV NC_013953				LdMNPV AF081810	LyxyNPV NC_013953
119	lef-1	118994<119698	123 (99.5)	134 (85.3)	122	lef-1	115363<116067	123 (99.5)	134 (85.3)	123	lef-1	118846<119550	123 (99.5)	134 (85.3)
120	LdORF124 like protein	119743>120141	124 (98.5)	133 (83.8)	123	LdORF124 like protein	116112>116516	124 (97.7)	133 (82.5)	124	LdORF124 like protein	119595>119993	124 (99.2)	133 (83.8)
	hr6	120144-120597				hr6	116518-116894				hr6	119994-120231		
121	egt	120601>122286	125 (98.5)	132 (90.7)	124	egt	116898>118583	125 (98.4)	132 (90.7)	125	egt	120223>121905	125 (99.6)	132 (91.7)
122	LdORF126 like protein	122308<122475	126 (96.3)		125	LdORF126 like protein	118605<118772	126 (96.3)		126	LdORF126 like protein	121927<122094	126 (100)	
123	LdORF127 like protein	122505>123086	127 (97.4)	129 (78.4)	126	LdORF127 like protein	118802>119383	127 (98.4)	129 (78.4)	127	LdORF127 like protein	122124>122708	127 (97.9)	129 (77.8)
124	ac17	123094>123774	128 (99.1)	128 (79.6)	127	ac17	119391>120071	128 (97.7)	128 (78.7)	128	ac17	122716>123396	128 (99.5)	128 (79.6)
125	LdORF129 like protein	123902<126631	129 (95.4)	127 (75.6)	128	LdORF129 like protein	120198<122798	129 (94.9)	127 (80.7)	129	LdORF129 like protein	123519<126149	129 (98.1)	127 (77)
126	Envelope protein	126766>128793	130 (99.2)	126 (96.1)	129	Envelope protein	122933>124960	130 (98.9)	126 (95.8)	130	Envelope protein	126278>128308	130 (99.7)	126 (96)
127	odv-e66	128897>130864	131 (99.8)	125 (92.2)	130	odv-e66	125061>127025	131 (99.8)	125 (92.2)	131	odv-e66	128409>130373	131 (99.8)	125 (92.5)
128	Zinc finger domain	130975>131238	132 (68.9)	124 (62.9)	131	Zinc finger domain	127136>127375	132 (88.8)	124 (65.4)	132	Zinc finger domain	130484>130726	132 (96.3)	124 (64.7)
129	Lyxy123 like protein	131460>131780		123 (87.5)	132	Lyxy123 like protein	127614>127934		123 (87.5)	133	Lyxy123 like protein	130982>131302		123 (87.5)
130	p24	131796>132464		122 (89.8)	133	p24	127950> 128621		122 (88.9)	134	p24	131318>131983		122 (89.7)
131	LdORF135 like protein	132424<132795	135 (100)	121 (80.5)	134	LdORF135 like protein	128581<128952	135 (99.1)	121 (81.4)	135	LdORF135 like protein	131943<132314	135 (100)	121 (80.5)
132	pp34/calyx	132910>133845	136 (99)	120 (89.7)	135	pp34/calyx	129066>130001	136 (99)	120 (89.7)	136	pp34/calyx	132429>133370	136 (100)	120 (89.4)
133	hypothetical	133934>134203		119 (78.1)	136					137	hypothetical	133449>133715		119 (77.9)
134	lef-2	134169>134813	137 (95.8)	118 (82.2)	137	lef-2	130328>130978	137 (96.7)	118 (80.5)	138	lef-2	133681>134325	137 (97.2)	118 (81.7)
135	LdORF138 like protein	134929>135804	138 (97.2)	117 (87.9)	138	LdORF138 like protein	131094>131969	138 (97.5)	117 (88.2)	139	LdORF138 like protein	134411>135286	138 (99.31)	117 (89.3)
136	iap-3	136056>136523	139 (97.4)	116 (82.5)	139	iap-3	132249>132716	139 (99.3)	116 (82.5)	140	iap-3	135540>136010	139 (98)	116 (81.4)

LdMNPV-H2 (MK264918)					LdMNPV-J2 (MK089451)					LdMNPV-T3 (MF311096)				
ORF	Name	Position	No. ORF (& aa identities)		ORF	Name	Position	No. ORF (& aa identities)		ORF	Name	Position	No. ORF (& aa identities)	
			LdMNPV AF081810	LyxyNPV NC_013953				LdMNPV AF081810	LyxyNPV NC_013953				LdMNPV AF081810	LyxyNPV NC_013953
137	ac106	136515<137258	140 (97.9)	115 (86.2)	140	ac106	132708<133451	140 (97.5)	115 (85.8)	141	ac106	136002<136736	140 (97.9)	115 (87.7)
138	LdORF141 like protein	137301<138929	141 (98.1)	114 (79.5)	141	LdORF141 like protein	133494<135122	141 (97.6)	114 (79.3)	142	LdORF141 like protein	136779<138407	141 (99.4)	114 (79.5)
139	LdORF142 like protein	138963<139346	142 (98.2)	113 (75.4)	142	LdORF142 like protein	135155<135532	142 (96.5)	113 (79.2)	143	LdORF142 like protein	138441<138824	142 (98.2)	113 (75.4)
140	pif-3	139355<139966	143 (99)	112 (88.5)	143	pif-3	135541< 136152	143 (98)	112 (87.5)	144	pif-3	138833<139444	143 (100)	112 (89)
141	LdORF144 like protein	140108<140449	144 (98.2)	111 (83.8)	144	LdORF144 like protein	136285< 136626	144 (97.3)	111 (83.8)	145	LdORF144 like protein	139590<139931	144 (100)	111 (83.8)
142	sod	140530>140994	145 (97.4)	110 (94.1)	145	sod	136707>137171	145 (98.7)	110 (94.1)	146	sod	140012>140476	145 (99.3)	110 (93.5)
	hr7a	140997-141473				hr7a	137173-137657				hr7a	140514-140780		
143	bro-l	141479>142522	146 (88.3)		146	bro-k	137659>139137	146 (64.8)		147	bro-l	140883> 141944	146 (97.7)	
144	rnr-r2a	142653<143732	147 (97.5)		147	rnr-r2a	139273<140352	147 (97.5)		148	rnr-r2a	142074< 143156	147 (98.3)	
145	rnr-r1	143839>145626	148 (97.6)		148	rnr-r1	140459>142246	148 (97.4)		149	rnr-r1	143263> 145050	148 (99.1)	
		145631-146236					142251-142744				hr7b	145110- 145467		
146	ctl-1	146240>146401	149 (100)		149	ctl-1	142748>142909	149 (100)		150	ctl-1	145537>145698	149 (100)	
147	bro-m	146567>147274	150 (95.6)		150	bro-l	143075>144490	150 (71.1)		151	bro-m	145869>146843	150 (68.5)	
148	LdORF151 like protein	147768<148280	151 (96.9)	143 (84.2)	151	LdORF151 like protein	144558<145616	151 (97.5)	143 (84.1)	152	LdORF151 like protein	147058<147564	151 (97.6)	143 (83.8)
149	LdORF152 like protein	148390>149142	152 (96.8)	144 (86.5)	152	LdORF152 like protein	145413>145925	152 (96.4)	144 (86.6)	153	LdORF152 like protein	147674>148426	152 (97.2)	144 (85.3)
150	bro-n	149188<149853	153 (67.2)		153	bro-m	145970<146935	153 (67.9)		154	bro-n	148492<149499	153 (91.1)	
	hr7c	149857-150565				hr7c	146937-147329				hr7c	149597-149928		
151	bro-o	150566<151573	153 (90.8)		154	bro-n	147331<148362	153 (87.2)						
152	bro-p	151940> 152938	154 (87.8)		155	bro-o	148408<149139			155	bro-o	150034>150771	154 (78.2)	
153					156	bro-p	149677>150375	154 (59.1)						
154	pif-1	153042> 154646	155 (95.5)	148 (92.1)	157	pif-1	150553>152154	155 (95.1)	148 (90.8)	156	pif-1	150950>152542	155 (98.3)	148 (90.2)

LdMNPV-H2 (MK264918)					LdMNPV-J2 (MK089451)					LdMNPV-T3 (MF311096)				
ORF	Name	Position	No. ORF (& aa identities)		ORF	Name	Position	No. ORF (& aa identities)		ORF	Name	Position	No. ORF (& aa identities)	
			LdMNPV AF081810	LyxyNPV NC_013953				LdMNPV AF081810	LyxyNPV NC_013953				LdMNPV AF081810	LyxyNPV NC_013953
155	fgf	155062<155919	156 (98.2)	149 (85.9)	158	fgf	152571<153425	156 (97.8)	149 (85.9)	157	fgf	152911<153765	156 (99.3)	149 (86.2)
156	alk-exo	155952<157214	157 (99.2)	150 (84.3)	159	alk-exo	153458<154720	157 (99.5)	150 (85.2)	158	alk-exo	153798<155060	157 (99.7)	150 (83.8)
157	ac18	157252<158373	158 (98.6)	151 (75.3)	160	ac18	154758<155879	158 (98.6)	151 (75.3)	159	ac18	155098<156219	158 (99.4)	151 (75.8)
158	LdORF159 like protein	158360>158716	159 (100)	152 (81.3)	161	LdORF159 like protein	155866>156222	159 (100)	152 (81.3)	160	LdORF159 like protein	156206>156562	159 (100)	152 (81.3)
159	vef-2	158750>161113	160 (95)	153 (94.2)	162	vef-2	156256>158622	160 (94.6)	153 (94.3)	161	vef-2	156587>158956	160 (97.4)	153 (94.8)
						hr8	158625-158948				hr8	158887-159193		
160	bro-q	161412>162368	161 (72.2)		163	bro-q	158950>159906	161 (71.3)	154 (89.2)	162	bro-p	159282>160304	161 (96.1)	
161	LdORF162 like protein	162514<162783	162 (97.8)	156 (88.5)	164	LdORF162 like protein	159997<160260	162 (100)	156 (87.3)	163	LdORF162 like protein	160450<160731	162 (97.8)	156 (76.6)
162	LdORF163 like protein	162907>163890	163 (92.1)	157 (67.3)	165	LdORF163 like protein	160384>161367	163 (92.1)	157 (67)	164	LdORF163 like protein	160863>161852	163 (97.5)	157 (67.6)
	hr1	163896-164643				hr1	161370-162168				hr1	161941-162455		

Supplementary File 2. Homologous repeated sequences were determined and aligned.

Hr1

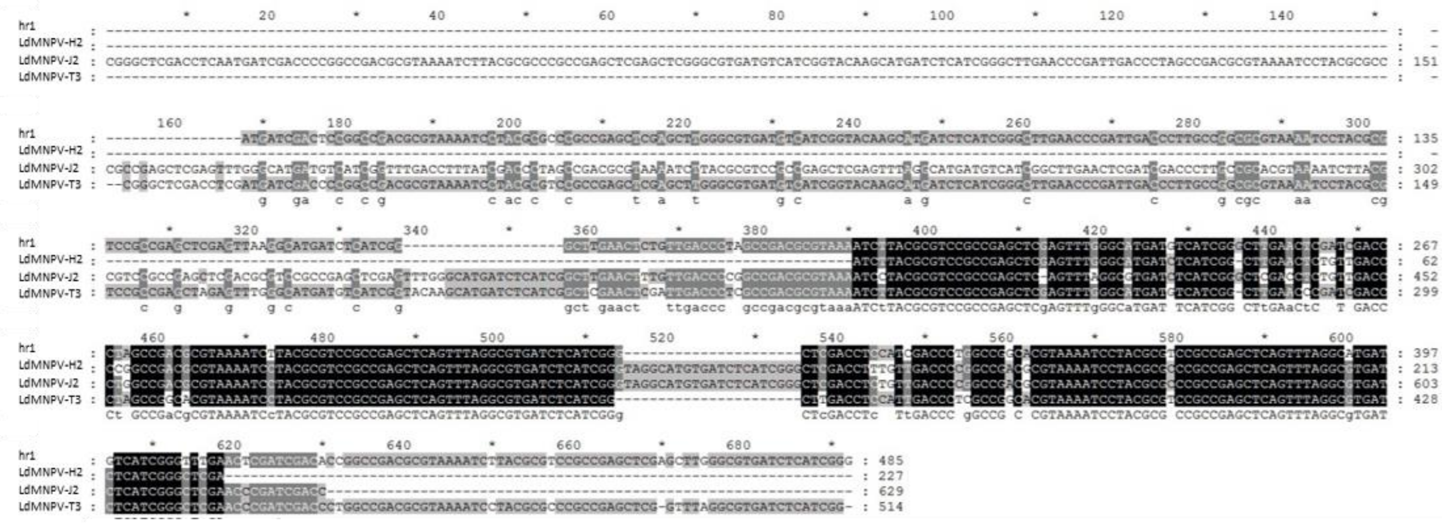

Hr2

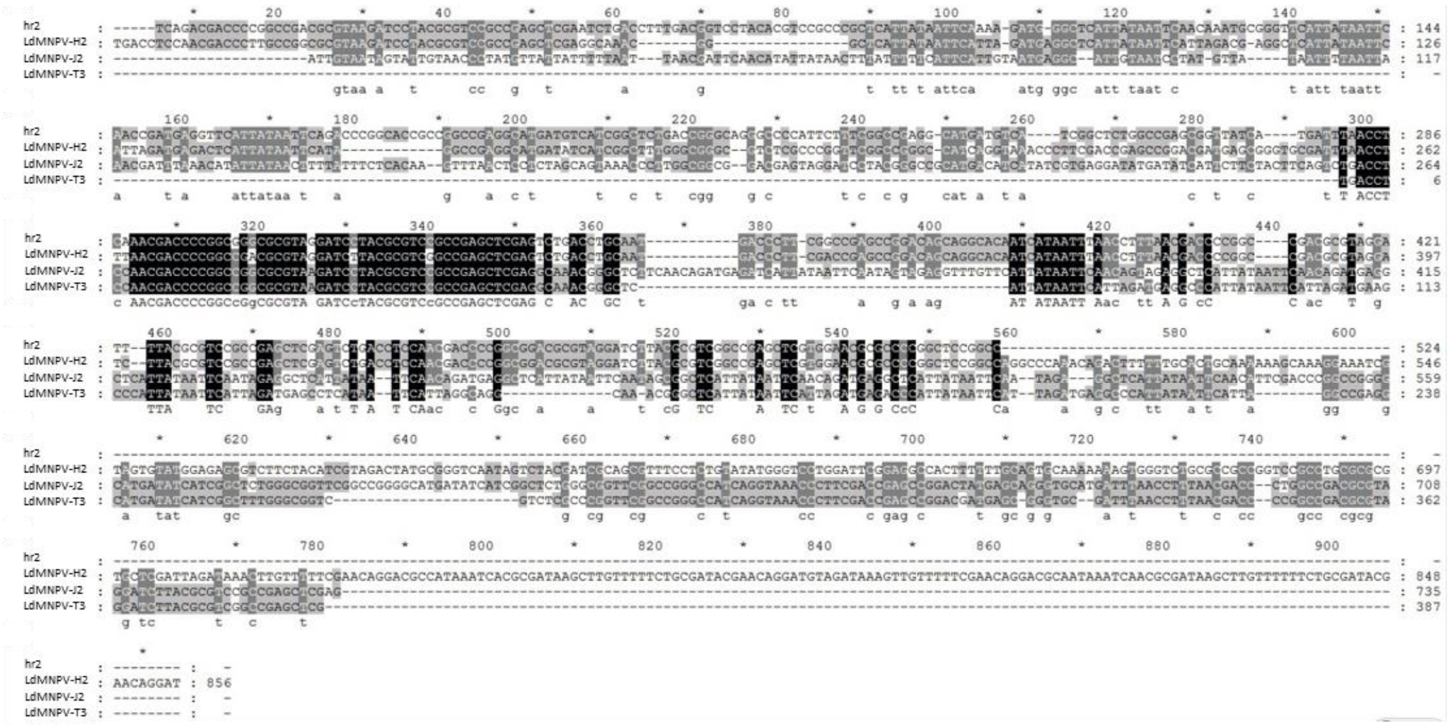

Hr3a

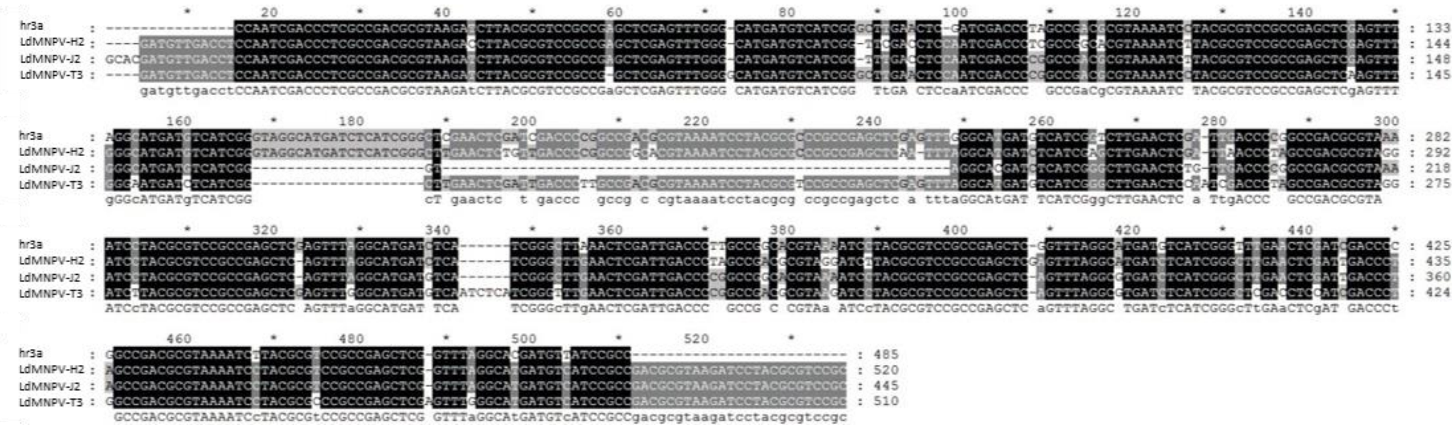

Hr3b

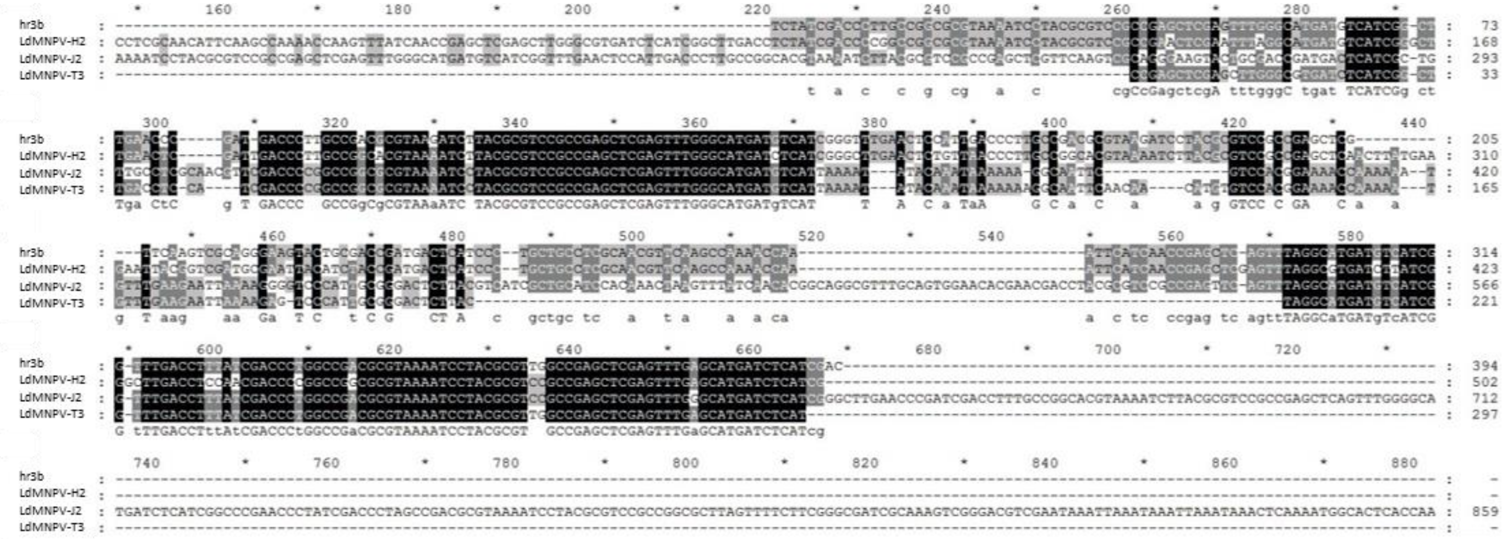

Hr3c

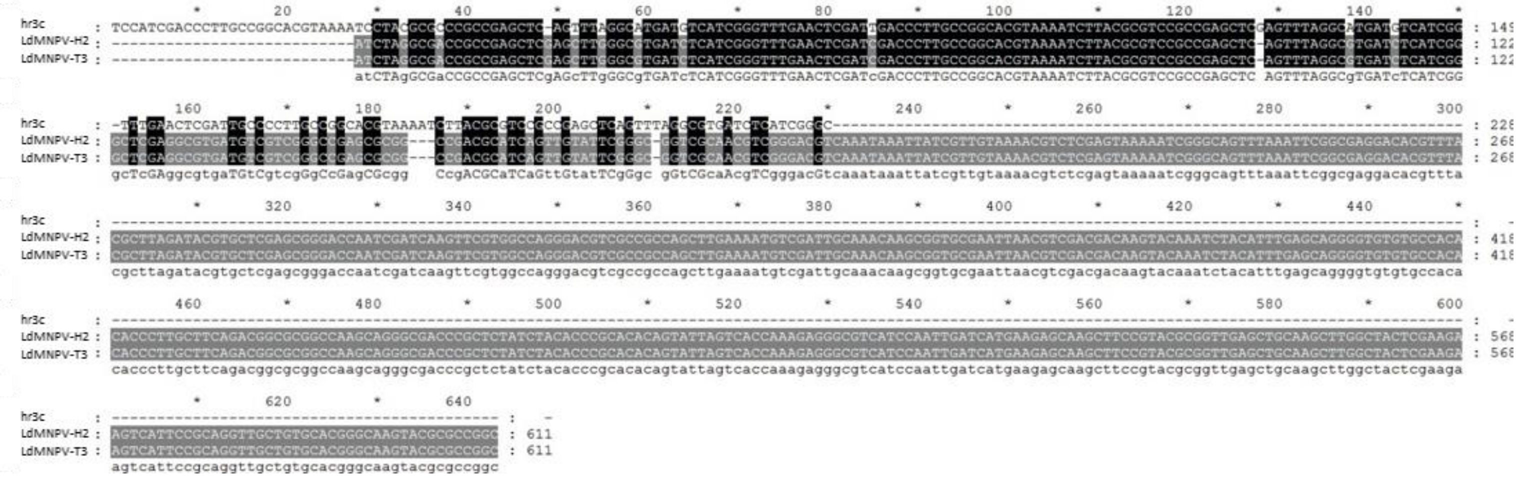

Hr4

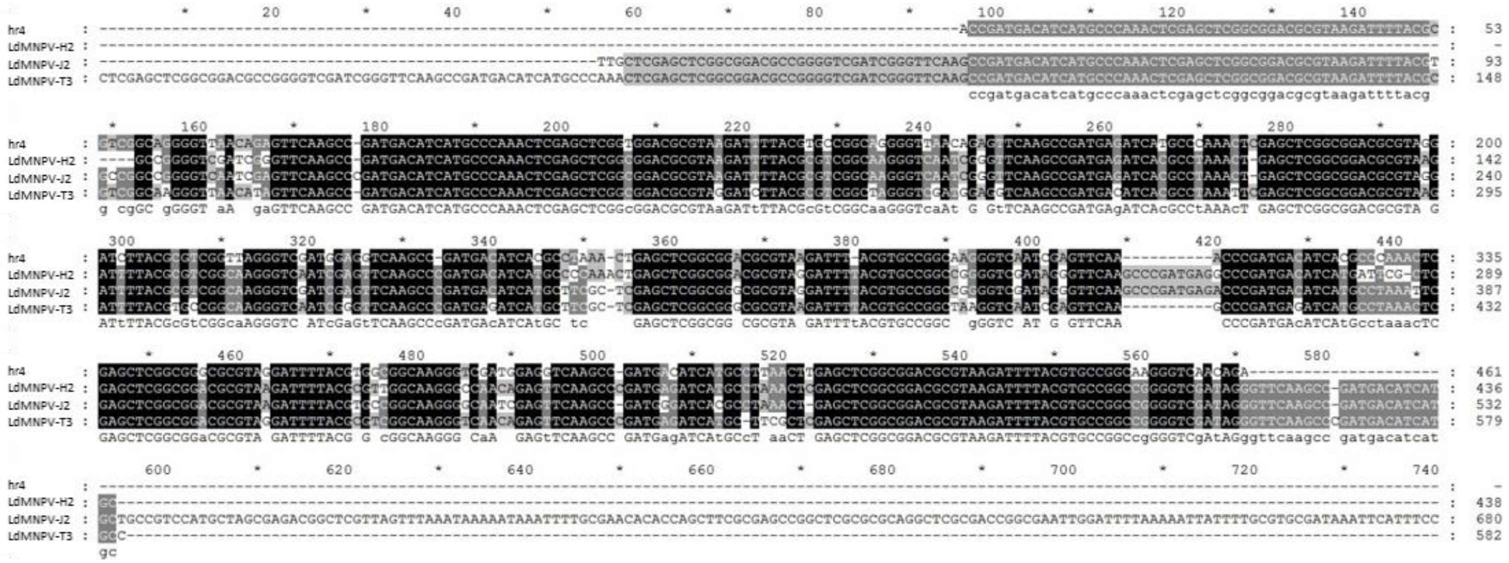

Hr5

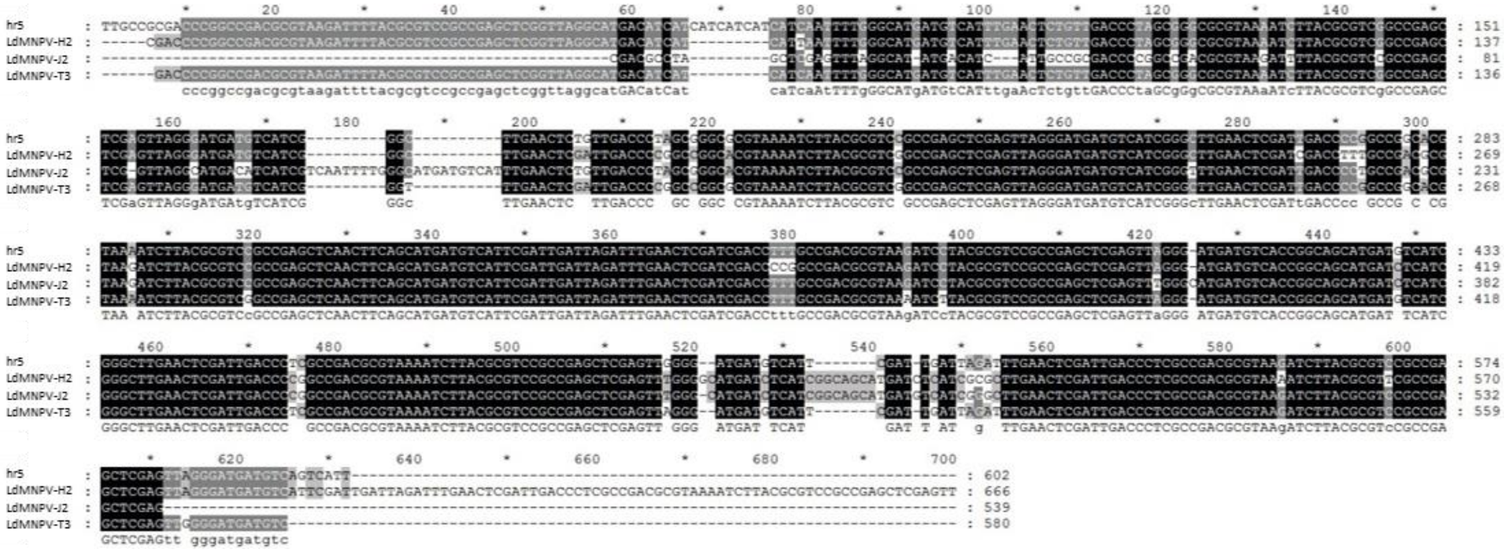

Hr6

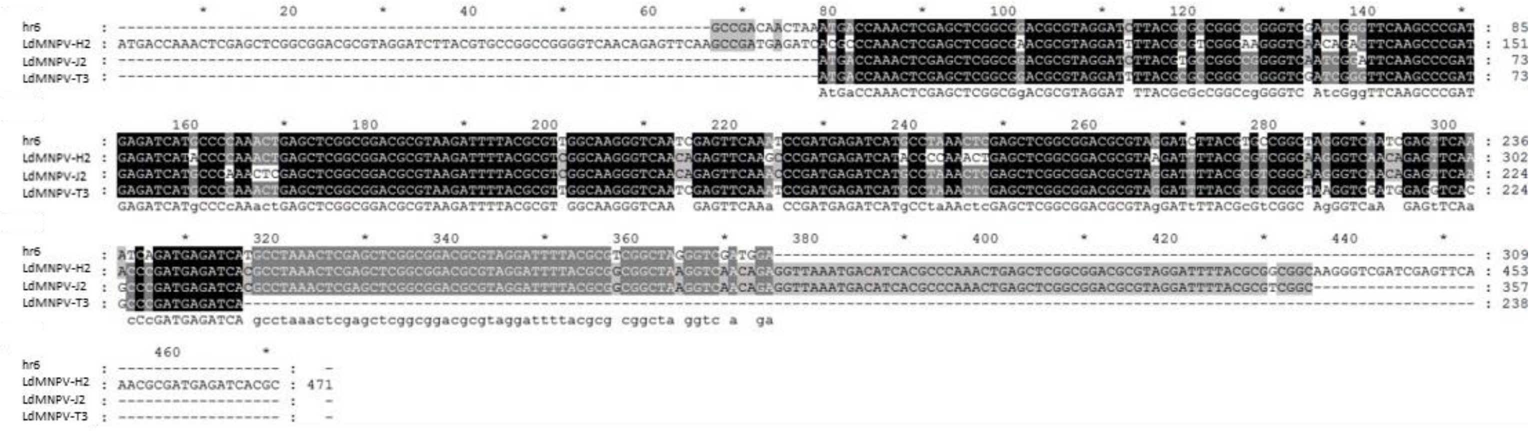

Hr7a

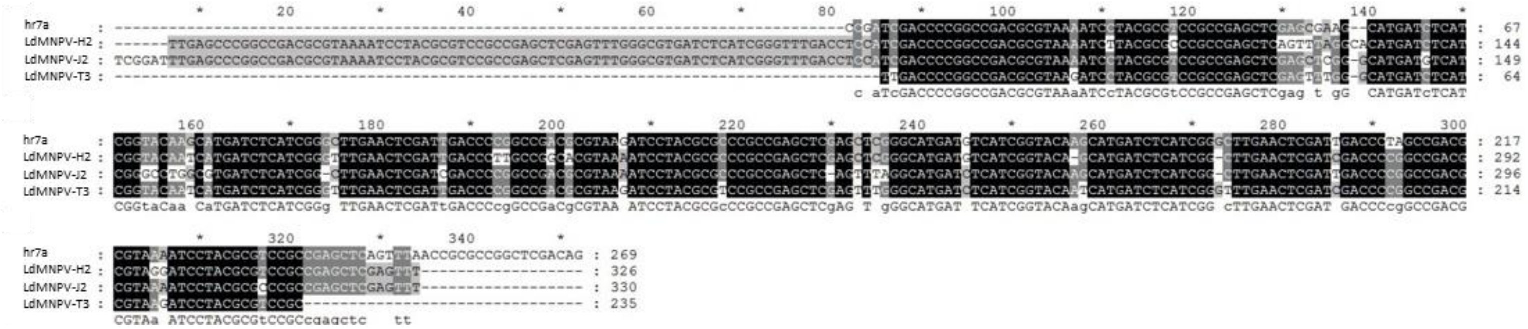

Hr7b

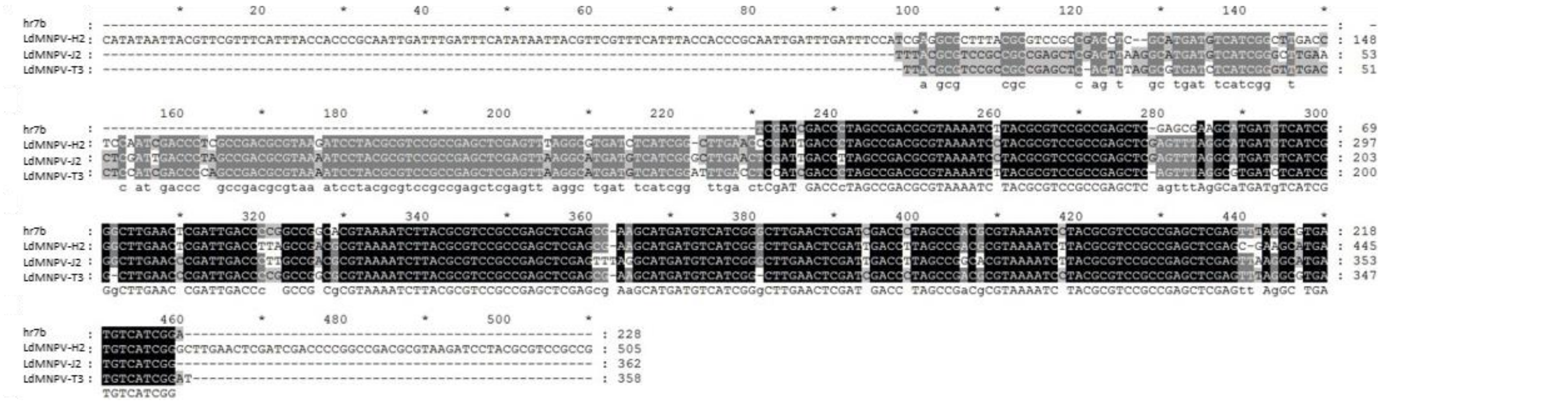

Hr7c

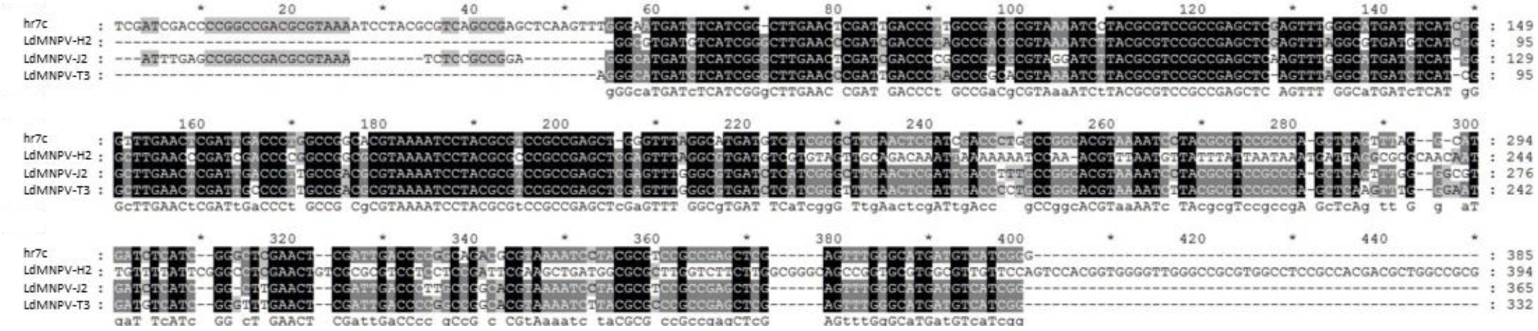

Hr8

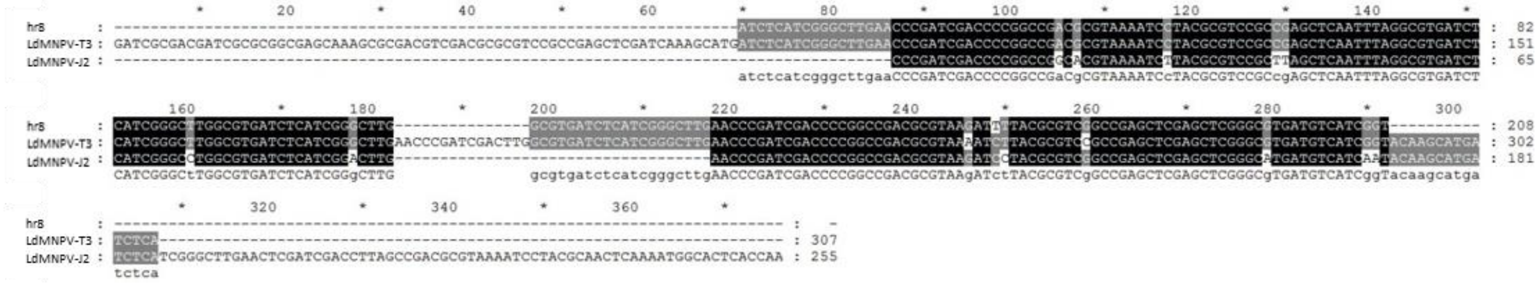

Supplement: Supplementary file 1 [file CG-24-146_SD1.pdf]
